# Workplace cessation support is associated with more abstinence in a workplace program in Hong Kong: A mixed-methods study

**DOI:** 10.18332/tid/156455

**Published:** 2022-12-23

**Authors:** Ziqiu Guo, Xue Weng, Alice Oi Sze Lau, Matthew Chak Hang Ng, Yongda Socrates Wu, Tai Hing Lam, Man Ping Wang

**Affiliations:** 1 School of Nursing, The University of Hong Kong, Hong Kong SAR, People's Republic of China; 2 Institute of Advanced Studies in Humanities and Social Sciences, Beijing Normal University, Zhuhai, People's Republic of China; 3 The Lok Sin Tong Benevolent Society Kowloon, Hong Kong SAR, People's Republic of China; 4 School of Public Health, The University of Hong Kong, Hong Kong SAR, People's Republic of China

**Keywords:** smoking cessation, mixed method, workplace smoking cessation support

## Abstract

**INTRODUCTION:**

We examined the association of workplace smoking cessation (SC) support from employers, in addition to SC interventions, and smoking abstinence.

**METHODS:**

Smoking employees (≥1 cigarette daily, aged ≥18 years) from companies of various industries joined a workplace SC program in Hong Kong. Self-reported past 7-day point prevalence abstinence was measured at follow-up at 6 months. We assessed 14 types of workplace SC support with higher scores (range: 0–14) indicating greater support. Multivariable logistic regression examined the prospective association between workplace SC support and smoking abstinence, adjusting for intention to quit, nicotine dependence, self-efficacy of quitting, and sociodemographic characteristics. Average marginal effects were calculated to test if the association between overall workplace SC support and self-reported past 7-day PPA at follow-up at 6 months was modified by subgroups. We also interviewed employers from different companies to explore their perspectives of providing workplace SC support, and the data were analyzed by thematic analysis.

**RESULTS:**

In 383 participants who received a heath talk, a self-help SC booklet, and 15 text messages, greater workplace SC support was associated with smoking abstinence (AOR=1.32; 95% CI: 1.08–1.61), including support for smoke-free environment (AOR=1.51; 95% CI: 1.08–2.11) and for SC attempts/actions (AOR=1.93; 95% CI: 1.21–3.07). The association did not differ by sex, age, intention to quit, nicotine dependence, company size or company type. Qualitative interviews found that employers provided workplace SC support to establish a good company image, cost-benefit considerations were important to the types of workplace SC support provided, and lack of SC knowledge was a barrier to providing workplace SC support.

**CONCLUSIONS:**

Greater workplace SC support was associated with more abstinence in a workplace SC program.

## INTRODUCTION

Smoking cessation (SC) interventions at the workplace can reach many smokers and attain high retention rate^1^. A systematic review of randomized controlled trials (RCTs) has shown that workplace SC interventions (behavioral and/or pharmacological approaches) effectively increase smoking abstinence^1^. Some employers offer SC support in addition to SC interventions, such as promoting SC activities and the use of SC services, adopting smoke-free workplace policy, and providing incentives^1-3^. Combined workplace SC intervention and workplace SC support offered by employers (e.g. health insurance benefits of SC treatment, incentives) effectively increase validated abstinence^4,5^. Additional SC support by employers in the workplace may further increase quitting outcomes of workplace SC intervention, but evidence is lacking. Our literature search in PubMed (by May 2022) using the search string: (‘workplace’ OR ‘worksite’) AND (‘intervention*’ OR ‘program*’ OR ‘support’) AND (‘smoking cessation’ OR ‘quit*’ OR ‘abstine*’), found no study that assessed abstinence outcomes of providing workplace SC support in addition to workplace SC interventions. We also found no definitions and tools for measuring workplace SC support.

Hong Kong, the most urbanized and developed city in China, has adopted stringent tobacco control measures, such as banning smoking in all indoor workplaces and public places, and providing free SC services. Despite having one of the lowest smoking prevalences in the developed world (9.5% daily cigarette use in 2021), most Hong Kong’s current smokers had never tried and did not want to quit smoking (66.2%)^6^. Nearly all (94.0%) of the current smokers who had not tried SC services before were unwilling to try these services^6^. Workplace-based SC programs are inadequate, and employees have long working hours (42 hours per week)^7^. As existing SC services have limited off-hour service, we organized and rigorously evaluated the first proactive outreach Smoking Cessation Program in Workplace (SCPW) in Hong Kong (SCPW-Phase I, 2012–2013). Details of the program have been reported elsewhere^8^. Using data from SCPW-Phase II (2015–2016), we examined the association of additional workplace SC support with smoking abstinence.

## METHODS

### Design

SCPW is funded by the Tobacco and Alcohol Control Office, Department of Health of Hong Kong SAR government, and organized by the Lok Sin Tong Benevolent Society, Kowloon (LST) in Hong Kong. Briefly, invitations were sent to companies selected from the list of companies (in alphabetic order) of the Care Company Scheme in Hong Kong or which had a good collaborating history with LST to initialize recruitments for SCPW-Phase II in 2015. Smoking employees of the participating companies were invited to attend a 1-hour health talk on smoking hazards, benefits of quitting, and methods to quit. Eligible participants were Chinese-speaking Hong Kong residents aged ≥18 years, smoking at least 1 cigarette per day, and staying in Hong Kong for the coming 12 months. They were invited to participate in the program after the health talk. All 725 participants received a 32-page self-help booklet at baseline and 15 text messages in the following 3 months (Supplementary file Table 1). They chose to join one of the three intervention groups (Group A: intensive counselling, n=22; Group B: brief face-to-face counselling, n=21; Group C: no additional intervention, n=523) after the health talk. Participants recruited individually (e.g. through booths in the community) or not available to attend the health talk joined Group D (n=159), which included telephone counselling as the key intervention. All participants provided written consent and were informed that they could withdraw from the program at any time without any interest loss.

**Figure 1:**
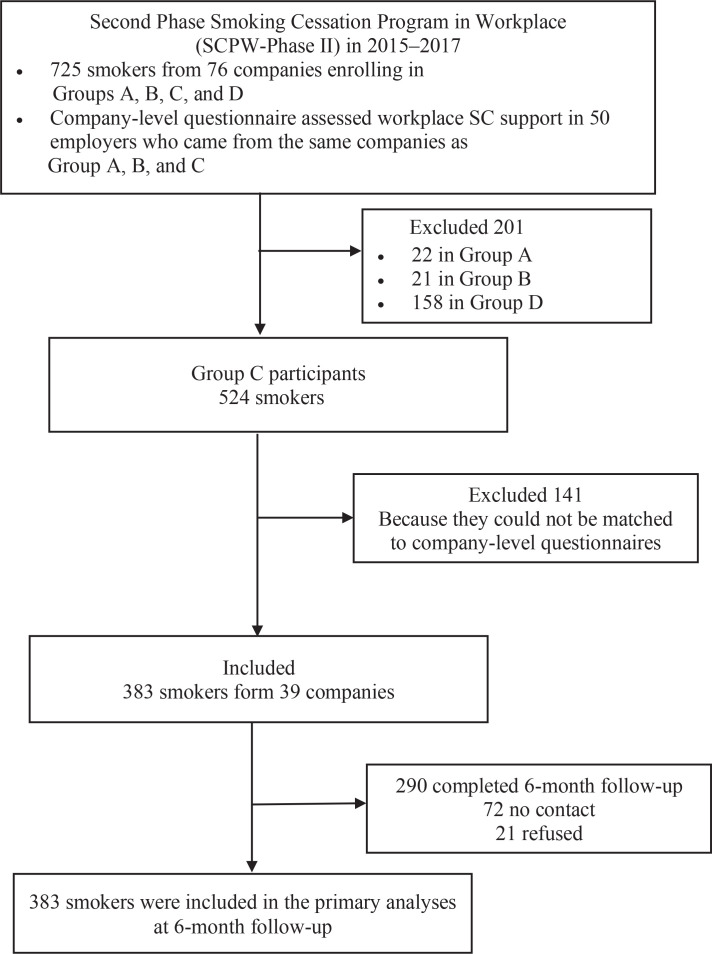
Sample selection procedure

The SCPW program also promoted workplace support for SC by organizing internal meetings with employers or managerial staff (hereafter as ‘employers’), which was assessed after completing the program. We invited employers to complete a company-level questionnaire as they were considered to be better aware of the company’s SC related policy and support than employees^3^. Fifty (of all 90 participating companies, 55.6%) companylevel questionnaires were completed. Only 383 Group C participants who could be matched with the 39 (of 50 completed, 78.0%) company-level questionnaires were included in the present analysis (Figure 1). Group D participants were excluded because information on workplace SC support was not available. We also excluded Group A and B participants (small sample size) because of different interventions (vs Group C) to avoid the potentially different effects from different interventions. The baseline characteristics of Group C participants included in the present analysis were comparable with the excluded Group A, B, D and C (Supplementary file Table 2).

### Measures

Participants self-administered the baseline questionnaire before the health talk. The measures included cigarette consumption and nicotine dependence, past quit attempt (yes/no), intention to quit (within 7 days/30 days/6 months/undecided), perceptions of quitting (importance, confidence, difficulty) were assessed on a scale of 0–10; higher scores indicating greater level^9^), and sociodemographic characteristics (sex, age, marital status, education level, and monthly household income). Nicotine dependence was assessed by the Heaviness of Smoking Index (HSI)^10^. HSI sums up the scores of 2 questions: the number of cigarettes smoked per day (0: 1–10; 1: 11–20; 2: 21–30; 3: >31) and time to the first cigarette after waking (0: after 60 minutes; 1: in 31–60 minutes; 2: in 6–30 minutes; 3: within 5 minutes). Higher scores indicate higher nicotine dependence. The total scores of HSI ≤2, 3–4, and 5–6, indicate low, moderate, and high levels of nicotine dependence, respectively. Telephone follow-up was conducted at 1 week, and at 1, 3, 6, and 12 months, after baseline to assess smoking cessation outcomes. In the present study, the main outcome was self-reported past 7-day point prevalence abstinence (PPA) at follow-up at 6 months.

Based on SCPW-Phase I outcomes and with further refinement according to the employers’ and SC experts’ comments, we designed one tool to measure workplace SC support by 14 types of support under three domains: providing incentives for successful quitters, providing support for SC attempts/actions, and smoke-free environment support (Supplementary file Table 3). Each type of SC support had a yes/no response. Affirmative responses were summed to derive a score of 0–14, with higher scores indicating greater workplace SC support. Specifically, providing incentives for successful quitters included cash, holidays, gifts, and public praises, with scores ranging from 0–4. Providing support for SC attempts/actions included paid time off for attending SC workshops, offering free healthy snacks in the pantry to replace smoking breaks, organizing sharing sessions on successful quitting experiences, and offering reimbursement for joining exercise programs/classes, with scores ranging from 0–4. Smoke-free environment support included email, notice, posters, other circulation on smoke-free information, setting desktop/screen saver as smoke-free logo, signing ‘Smoke-free contact’ with employees, setting up ‘Smoke-free workplace’ committee, posters on successful quitting cases, organizing ‘smoke-free slogan’ competition, with scores ranging from 0–6. We did not include employer-offered insurance coverage on SC treatment as it was rarely provided, and SC treatments are free in Hong Kong.

Company size was categorized by the number of personnel into: small (≤10), medium (11–100), and large (≥100). Occupation types were recoded based on the Standard Occupational Classification (SOC) system and then classified into white-collar, blue-collar, and services^11^.

### Statistical analysis

Stata 15.1 (Stata Crop LP, College Station, TX, USA) was used. Multivariable logistic regressions yielded adjusted odd ratios (AORs) for self-reported past 7-day PPA at follow-up at 6 months by the level of workplace SC support [overall workplace SC support (0–14) and each of the three domains: providing incentives for successful quitters (0–4), providing support for SC attempts/actions (0–4), and smoke-free environment support (0–6)] and participants’ baseline characteristics (sex, age, company size and type, intention to quit, nicotine dependence, and perceptions of quitting). We used multiple imputation by chained equation to impute missing values in self-reported past 7-day PPA at follow-up at 6 months and participants’ baseline characteristics under the missing-at-random assumption^12^. Multiple imputation is a commonly used approach to handle missing data by creating several (n=50) imputed datasets and combining results from analyses in each imputed dataset following Rubin’s rule^13^. The imputation model was based on variables included in the main analysis and additional ones that are predictors of quitting in the literature. Including these variables in the imputation model would lower the chance of violating the missing-at-random assumption, increase statistical power (large sample size), and reduce selection bias. To test if the association between overall workplace SC support and self-reported past 7-day PPA at follow-up at 6 months was modified by subgroups (i.e. sex, age, quit intention, nicotine dependence, company size, and company type), average marginal effects (AMEs)^14^ by subgroups were calculated. AMEs are the changes in participants’ predicted probability of self-reported past 7-day PPA at 6 months in relation to per score change in overall workplace SC support. A significant difference in AMEs between subgroups of a characteristic (e.g. male vs female) suggests effect modification.

### Workplace SC support: qualitative interviews of employers

After completing the SCPW program, we individually interviewed 9 employers from different companies (5 from the property management industry) using a semi-structured interview guide. Characteristics of the interviewees are given in Supplementary file Table 4. The interviews aimed to explore employers’ perspectives and experience with the program, including providing workplace SC support. In the present study, we only presented results related to providing workplace SC support. Interviewees were first asked to describe the workplace SC support they had provided. Their perspectives and experiences of providing workplace SC supports were further explored using open-ended questions (e.g. ‘What factors would you consider when deciding to provide workplace SC support?’, ‘What were the difficulties of providing workplace SC support?’).

All interviews were audio-recorded and transcribed verbatim. Thematic analysis described by Braun and Clarke^15^ was done. Researcher (ZG) read all transcripts carefully to generate preliminary thoughts on the data, then coded the passages related to the question. Codes that shared similar meanings were collated as initial themes. After reviewing and refining these initial themes, the final themes were defined and named.

## RESULTS

### Quantitative results

Table 1 shows that 94.0% of participants (n=383) were male, 29.0% were aged ≥50 years, and 62.1% were married or cohabiting. Most participants had secondary education (60.3%), and 44.9% had a monthly household income of HK$30000 or above (US$1=HK$7.8). On average, participants smoked for 17.80 years (SD=9.92) and 14.90 cigarettes (SD=17.35) per day. Most participants had light (53.9%) or moderate (41.8%) levels of nicotine dependence. A quarter of participants had never made quit attempts before, and 79.8% had no intention to quit. Participants perceived moderate importance (6.63 ± 3.05), confidence (5.68 ± 2.98), and difficulty (6.11 ± 3.11) of quitting.

**Table 1:** Participants’ sociodemographic and smoking-related characteristics at baseline (N=383)

*Characteristics*	*n (%)*
**Sex**	
Male	360 (94.0)
Female	23 (6.0)
**Age** (years)	
≤29	56 (18.1)
30–39	78 (25.2)
40–49	86 (27.7)
≥50	90 (29.0)
**Marital status**	
Single	98 (32.0)
Married/cohabiting	190 (62.1)
Widowed/separated/divorced	18 (5.9)
**Education level**	
Primary or lower	32 (10.3)
Secondary	188 (60.3)
Tertiary	92 (29.5)
**Monthly household income** (HK$)^a^	
≤19999	55 (26.8)
20000–29999	58 (28.3)
≥30000	92 (44.9)
**Smoking**	
Years of smoking, mean ± SD	17.80 ± 9.92
Daily cigarette consumption, mean ± SD	14.90 ±17.35
**Nicotine dependence^b^**	
Light (≤2)	165 (53.9)
Moderate (3–4)	128 (41.8)
Heavy (5–6)	13 (4.3)
**Past quit attempts**	
No	89 (25.0)
Yes	267 (75.0)
**Intention to quit**	
Within 7 days	19 (6.0)
Within 30 days	23 (7.3)
Within 6 months	22 (6.9)
Not decided yet	253 (79.8)
**Perception of quitting^c^,** mean ± SD	
Importance	6.63 ± 3.05
Confidence	5.68 ± 2.98
Difficulty	6.11 ± 3.11

a US$1=HK$7.8.

b Measured by Heaviness of Smoking Index (HSI) score with range 0–6; HSI ≤2 light, HSI 3–4 moderate, HIS 5–6 heavy.

c Score: 0–10, higher scores indicating higher perceived importance, higher confidence, and higher difficulty of quitting.

Table 2 shows that 91.9% of participants worked in large companies; 56.9% were blue-collar workers, and 40% were white-collar workers. The mean score of the overall workplace SC support was 2.30 ± 1.68 (out of 14). The mean score for providing an incentive to successful quitters, providing support for SC attempts/actions, and smoke-free environment support was 0.14 ± 0.44 (out of 4), 0.91 ± 0.71 (out of 4), and 1.25 ± 0.91 (out of 6), respectively. Nearly all (95.8%) participants were offered one or more smoke-free environment supports, 74.2% were offered one or more supports for SC attempts/actions, but only 10.2% were offered one or more incentive supports.

**Table 2:** Characteristics of companies and workplace smoking cessation support (N=383)

*Characteristics*	*n (%)*
**Company size^a^** (personnel)	
Small (1–10)	3 (0.9)
Medium (11–100)	23 (7.1)
Large (>100)	296 (91.9)
**Occupation type^b^**	
White-collar	153 (40.0)
Blue-collar	218 (56.9)
Service	12 (3.1)
**Overall, workplace smoking cessation support (0–14),** mean ± SD	2.30 ± 1.68
**Incentive for successful quitters (0–4),** mean ± SD	0.14 ± 0.44
Cash	5 (1.3)
Holidays	7 (1.8)
Gifts	15 (3.9)
Public praises	25 (6.5)
Any of the above	39 (10.2)
**Support for smoking cessation attempts/actions (0–4),** mean ± SD	0.91 ± 0.71
Paid time off for attending smoking cessation workshops	254 (66.3)
Offered free healthy snacks in the pantry to replace smoking breaks	73 (19.1)
Organized sharing sessions on successful quitting experiences	16 (4.2)
Offered reimbursement for joining exercise program/classes	5 (1.3)
Any of the above	284 (74.2)
**Smoke-free environment support (0–6),** mean ± SD	1.25 ± 0.91
Email, notice, posters, other circulation on smoke-free information	354 (92.4)
Set desktop/screen saver as smoke-free logo	9 (2.35)
Sign ‘Smoke-free contact’ with employees	15 (3.92)
Set up ‘Smoke-free workplace’ committee	53 (13.8)
Poster on successful quitting cases	31 (8.1)
Organized ‘smoke-free slogan’ competition	17 (4.4)
Any of the above	367 (95.8)

a Missing data were excluded in the analysis.

b White-collar: sales and related occupations; management occupations; business and financial operations occupations; educational instruction and library occupations; arts, design, entertainment sports and media occupations; computer and mathematical science occupations. Blue-collar: installation maintenance and repair occupations; construction and extraction occupations; transportation and material moving occupations; production occupations. Service: food preparation and serving related occupations; building and grounds cleaning and maintenance occupations; healthcare support occupations; personal care and service occupations.

The self-reported past 7-day PPA at follow-up at 6 months was 20.9% (80/383). Table 3 shows that greater workplace support was associated with self-reported past 7-day PPA at follow-up at 6 months (overall: AOR=1.32; 95% CI: 1.08–1.61; support for SC attempts/actions: AOR=1.93; 95% CI: 1.21–3.07, smoke-free environment support: AOR=1.51; 95% CI: 1.08–2.11).

**Table 3:** The associations of workplace SC support, participants’ characteristics with self-reported smoking abstinence at 6 months follow-up

*Factors*	*AOR (95% CI) for self-reported 7-day PPA at 6 months*			
	*Incentives for successful quitters (0–4)*	*Support for smoking cessation attempts/actions (0–4)*	*Smoke-free environment support (0 – 6)*	*Overall workplace SC support (0–14)*
**Workplace SC support^a^**	1.59 (0.60–4.21)	1.93 (1.21–3.07)*	1.51 (1.08–2.11)*	1.32 (1.08–1.61)*
**Sex**				
Male (Ref.)	1	1	1	1
Female	0.83 (0.21–3.22)	0.87 (0.22–3.46)	0.83 (0.21–3.25)	0.80 (0.20–3.19)
**Age** (years)				
≤29 (Ref.)	1	1	1	1
30–39	0.76 (0.27–2.15)	0.82 (0.29–2.35)	0.81 (0.29–2.29)	0.81 (0.29–2.29)
40–49	0.91 (0.34–2.47)	0.87 (0.32–2.41)	0.89 (0.33–2.42)	0.91 (0.33–2.47)
≥50	0.99 (0.34–2.96)	1.08 (0.36–3.22)	1.06 (0.36–3.16)	1.10 (0.37–3.27)
**Company size**				
Small/medium (≤100) (Ref.)	1	1	1	1
Large (>100)	2.09 (0.43–10.08)	1.75 (0.47–6.57)	1.71 (0.45–6.48)	2.16 (0.53–8.77)
**Nicotine dependence** (HSI)				
Light (≤2) (Ref.)	1	1	1	1
Moderate/heavy (3–6)	0.50 (0.23–1.11)	0.48 (0.22–1.07)	0.49 (0.22–1.09)	0.49 (0.22–1.09)
**Intention to quit within 30 days**				
No (Ref.)	1	1	1	1
Yes	2.54 (1.04–6.22)*	2.73 (1.10–6.80)*	2.63 (1.06–6.52)*	2.67 (1.08–6.61)*
**Occupation type**				
White-collar (Ref.)	1	1	1	1
Service	0.91 (0.16–5.21)	0.82 (0.14–4.73)	1.56 (0.27–8.89)	1.04 (0.18–6.00)
Blue-collar	0.52 (0.26–1.06)	0.56 (0.27–1.16)	0.63 (0.30–1.36)	0.64 (0.30–1.36)
**Perception of quitting**				
Importance of quitting	1.00 (0.87–1.15)	0.99 (0.85–1.14)	1.00 (0.87–1.16)	0.99 (0.86–1.15)
Confidence of quitting	1.19 (1.02–1.39)*	1.22 (1.04–1.43)*	1.21 (1.04–1.41)*	1.22 (1.04–1.43)*
Difficulty of quitting	0.90 (0.80–1.01)	0.91 (0.81–1.03)	0.90 (0.80–1.02)	0.91 (0.80–1.02)

a The corresponding type of workplace SC support included in each of model is shown in the 2nd row.

* p<0.05; **p<0.01; ***p<0.001. AOR: adjusted odds ratio. PPA: point prevalence abstinence.

Table 3 also shows that participants with intention to quit within 30 days were associated with self-reported past 7-day PPA at 6 months with AORs ranging from 2.78 to 2.98 (all p<0.05). Participants with higher quitting confidence reported higher odds of self-reported past 7-day PPA at 6 months (AOR: 1.19 to 1.22, all p<0.05).

We found a moderate but insignificant association between providing incentives for successful quitters and self-reported past 7-day PPA at 6 months (AOR=1.59; 95% CI: 0.60–4.21). No effect modification by sex, age group, intention to quit, nicotine dependence, company size, or occupation type were observed (Table 4).

**Table 4:** Increases in participants’ predicted probability (average marginal effects) of self-reported past 7-day PPA at 6 months, given one score increase in overall workplace SC support, by participants characteristics

*Factors*	*Self-reported past 7-day PPA at 6 months % (95% CI)*
**Sex**	
Male	4.49 (1.41–7.56)
Female	4.15 (1.41–7.55)
**Age** (years)	
≤29	4.81 (1.37–8.24)
30–39	4.05 (1.05–7.04)
40–49	4.54 (1.44–7.63)
≥50	4.54 (1.26–7.82)
**Intention to quit within 30 days**	
No	4.25 (1.34–7.17)
Yes	5.73 (1.66–9.80)
**Nicotine dependency** (HSI)	
Light (≤2)	5.21 (1.67–8.76)
Moderate/heavy (3–6)	3.56 (0.89–6.22)
**Company size**	
Small/medium (≤100)	3.80 (1.02–6.58)
Large (>100)	4.55 (1.39–7.72)
**Occupation type**	
White-collar	4.79 (1.66–7.92)
Blue-collar	4.71 (0.96–8.47)
Service	4.22 (1.16–7.28)

Based on the multivariable logistic model for overall SC support in Table 3. Average marginal effects (AMEs) are the average changes in predicted probability of outcomes in relation to one score increased in overall workplace SC support (0–14) when other baseline characteristics in the model remained unchanged. SC: smoking cessation. PPA: point prevalence abstinence.

### Qualitative results

We identified three themes from interviews with employers: 1) establish a good company image; 2) cost-benefit consideration; and 3) lacking SC knowledge as a barrier to providing workplace SC support.

Most interviewees reported that they were willing to provide workplace SC support because they wanted to establish a good company image:

*‘The reason for providing workplace SC support was company image. We can’t let customers see our colleagues smoking on the streets.’* (Interviewee 8, male, aged 35 years, from sales industry)

Interviewees reported the cost-benefit consideration when deciding to provide which types of workplace SC support. They preferred to provide types of support that are beneficial but cost less. Some interviewees reported that providing incentives required the directors to approve the budget, which depended on their perceived benefits of helping smoking employees quit:

*‘We are customer severing industry. We need to consider whether providing the workplace SC support would affect our service. If the workplace SC support would not cost much time but improve our service, we would provide (workplace SC support).’* (Interviewee 9, male, aged 37 years, from property management industry)

*‘Providing incentive involved budget, which was depended on if directors thought SC was important.’* (Interviewee 5, female, from property management industry)

Interviewees also reported a lack of professional SC-related knowledge was the barrier to providing workplace SC support:

*‘It was difficult for us to design workplace SC programs to help employees to quit because we were not experts of SC, we lacked the professional knowledge and didn’t know what should do.’* (Interviewee 9, male, aged 37 years, from property management industry)

## DISCUSSION

To our knowledge, this is the first study that assessed the effect of offering workplace SC support on smoking abstinence in addition to workplace SC intervention. We found that greater workplace SC support (mainly support for smoke-free environment and for SC attempts/actions) was associated with more abstinence in smoking employees who were receiving a workplace SC intervention. The association did not differ by sex, age, intention to quit, nicotine dependence, company size or company type. Qualitative interviews in employers found that establishing a good company image was the main reason of offering SC support at the workplace. But cost-benefits considerations dominated the type of workplace SC support provided. Employers perceived the lack of professional SC knowledge as the main barrier to offering SC support.

Previous studies have shown that employers offered workplace SC support, such as insurance coverage on SC services and financial incentives, was associated with higher abstinence rates without providing SC interventions^2,3^. Our study adds to the literature by showing workplace SC support was associated with more abstinence even when smoking employees were receiving an effective SC intervention (30% of participants self-reported abstinence rate at 6 months since receiving a health talk, a self-help SC booklet and 15 SMS in SCPW-Phase I)^8^. This was due to workplace SC support in our study consisting of support for SC attempts/actions and smoke-free environment, which was found to be directly associated with smoking abstinence. Offering workplace SC support such as promoting smoke-free environment may encourage abstinence by de-normalizing smoking in employees^16^. A recent national representativeness survey in China found that exposure to anti-tobacco information was associated with quit attempts^17^. These may partly explain the association between workplace SC support and abstinence in the SC program. Our finding suggested that workplace SC support could be added to strengthen the effect of workplace SC interventions. Meanwhile, future studies that aim to compare SC interventions situated in the different workplaces may need to consider the confounding caused by the potential involvement of workplace SC support.

We found that providing incentives to the successful quitter was not associated with smoking abstinence at 6 months in our study. This may be due to this study including different types of incentives, and some of the incentives (e.g. public praise in a SC ceremony) were not effective for SC. In contrast, increasing evidence has shown that offering contingent financial incentives (i.e. offering cash/vouchers after quitting successfully) was associated with smoking abstinence in workplace settings across different countries^1,5^. Most studies adopted financial incentives, monetary or vouchers, as incentives^18^, the effect of providing holidays, gifts, and public praise as incentives for smoking abstinence in the workplace was understudied and needed to be examined further. Another reason for the null effect could be only a few participants (n=39) were offered incentives support. Future studies should have an adequately powered sample size as indicated by a wide 95% CI (OR=1.59; 95% CI: 0.60–4.21) in our study.

Cost-benefits considerations were crucial to deciding the types of workplace SC support offered. This is consistent with our previous survey’s findings that employers who perceived a higher impact of smoking on companies were more likely to promote SC in the workplace^19^. A qualitative study in employers also reported that cost-benefits were considered when offering financial incentives and group-based training as workplace support for their smoking employees^20^. We found employers perceived that the consequence of smoking in employees was mainly on company image. None of the interviewed employers mentioned the consequence of smoking on productivity, which was similar with our previous finding that fewer employers perceived smoking impact productivity loss^19^. A recent meta-analysis in working populations has shown that smoking was associated with a 31% increased risk and 2.89 days (per year) of sickness absence^21^. Modeling studies using national-representative data of Australia and Malaysia also found smoking resulted in a loss of productivity^22,23^. Former smokers were found to have increased the workplace productivity and decreased absenteeism compared with continuing smokers within 1–4 years of cessation^24^. Apart from improving the image of the company, supporting smoking employees quit could also increase the productivity of these employees, something that employers in Hong Kong are not fully aware of. More efforts, such as providing employers information on the cost-benefits of offering SC support, are warranted to promote workplace SC support. Rigorous cost-effectiveness or cost-utility analysis of offering SC support at the workplace in Hong Kong are further needed to obtain evidence and substantiate the provision of workplace support.

### Limitations

This study had some limitations. First, employers and smoking employees who joined the SCPW may be more concerned about smoking, which might limit the generalities of our findings. Second, most participants were male. But it was consistent with a much higher smoking prevalence in males (16.7%) than in females (3.0%) in Hong Kong^6^. Third, lack of biochemical validation for quitting might result in over-reporting of successful quit. Fourth, workplace SC support in our study was reported by employers, and employees might not receive or be aware of this support provided by the workplace. The agreement between employers’ and employees’ reported workplace support and associations between employees’ self-reported and objectively validated workplace SC support and smoking abstinence should be further studied. Lastly, our outcome was mainly based on large size companies’ information on workplace SC support, where the priority of workplace tobacco control was different from small companies^25^. Further study in different sizes of companies to assess the effect of workplace SC support is warranted.

## CONCLUSIONS

This study found greater workplace SC support was associated with more smoking abstinence in a workplace SC program. Workplace SC support could potentially be integrated within SC programs.

## Data Availability

The data supporting this research are available from the authors on reasonable request.
