# Supplementary material for: Workplace cessation support is associated with more abstinence in a workplace program in Hong Kong: A mixed-methods study
Source: Tob Induc Dis. 2022 Dec 23;20:114. doi: 10.18332/tid/156455 (PMC9782256; doi:10.18332/tid/156455)
Supplement: Supplementary file 1 [file TID-20-114-s1.pdf]

Table 1. Detail intervention contents for participants who joined Second Phase Smoking Cessation Program in Workplace (SCPW-Phase II).

Group	N	Intervention contents
A	22	1h health talk + 4h intensive workshop + self-help SC booklet + 15 SMS
B	21	1h health talk + 1h face-to-face counselling + self-help SC booklet + 15 SMS
C	524	1h health talk + self-help SC booklet + 15 SMS
D	158	45 minutes phone counselling + self-help SC booklet + 15 SMS

SC: smoking cessation
SMS: short messaging service

Table 2. Baseline characteristics of participants in Group C included in the analysis (n=383), and the excluded Group A (n=22), B (n=21), and D (n=158), and Group C (n=141).

	Group C included in analysis (%)	Group A (%) [c]	Group B (%) [c]	Group C excluded in analysis (%) [d]	Group D (%) [e]
Sociodemographic					
Sex					
Male	360 (94.0)	19 (86.4)	18 (85.7)	130 (92.2)	141 (89.2)
Female	23 (6.0)	3 (13.6)	3 (14.3)	11 (7.8)	17 (10.8)
Age (years)					
≤ 29	56 (18.1)	4 (20.0)	2 (9.5)	19 (15.5)	8 (5.6)
30 - 39	78 (25.2)	8 (40.0)	6 (28.6)	32 (26.0)	38 (26.4)
40 - 49	86 (27.7)	4 (20.0)	3 (14.3)	34 (27.6)	45 (31.3)
≥ 50	90 (29.0)	4 (20.0)	10 (47.6)	38 (30.9)	53 (36.8)
Marital status					
Single	98 (32.0)	6 (30.0)	5 (23.8)	31 (27.0)	31 (20.5)
Married/cohabited	190 (62.1)	12 (60.0)	13 (61.9)	75 (65.2)	109 (72.2)
Widow/separated/divorced	18 (5.9)	2 (10.0)	3 (14.29)	9 (7.8)	11 (7.3)
Education					
Primary or below	32 (10.3)	0 (0.0)	4 (20.0)	17 (14.3)	15 (10.9)
Secondary	188 (60.3)	13 (65.0)	13 (65.0)	78 (65.6)	106 (76.8)
Tertiary	92 (29.5)	7 (35.0)	3 (15.0)	24 (20.2)	17 (12.3)
Monthly household income (HK$) [a]					
≤ 19999	55 (26.8)	3 (18.8)	9 (69.2)	35 (38.9)	31 (34.1)
20000-29999	58 (28.3)	3 (18.8)	0 (0.0)	24 (26.7)	20 (22.0)
≥ 30000	92 (44.9)	10 (62.5)	4 (30.8)	31 (34.4)	40 (44.0)
Smoking					
Years of smoking (mean ± SD)	17.80 ± 9.92	20.13 ±9.52	22.67 ± 12.47	22.54 ± 11.98	23.81 ± 11.60
Daily cigarette consumption (mean ± SD)	14.90 ±17.35	16.43 ± 13.10	15.43 ± 8.23	15.74 ± 26.45	14.74 ± 9.43
Nicotine dependency [b]					
Light (≤2)	165 (53.9)	10 (50.0)	8 (38.1)	69 (56.6)	79 (52.3)
Moderate (3-4)	128 (41.8)	8 (40.0)	13 (61.9)	44 (36.1)	62 (41.1)
Heavy (5-6)	13 (4.3)	2 (10.0)	0 (0.0)	9 (7.4)	10 (6.2)
Past quit attempt					
No	89 (25.0)	4 (19.1)	3 (14.3)	23 (17.4)	29 (18.4)
Yes	267 (75.0)	17 (81.0)	18 (85.7)	109 (82.6)	129 (81.7)
Intention to quit					
Within 7 days	19 (6.0)	3 (16.7)	2 (10.5)	8 (7.4)	11 (8.0)
Within 30 days	23 (7.3)	5 (27.8)	1 (5.3)	10 (9.3)	13 (9.5)
Within 6 months	22 (6.9)	1 (5.6)	2 (10.5)	5 (4.6)	12 (8.8)
Not decided yet	253 (79.8)	9 (50.0)	14 (73.7)	85 (78.7)	101 (73.7)

[a] US $ 1 = HK$ 7.8

[b] Measured by Heaviness of Smoking Index (HSI) with range 0-6, HSI ≤ 2 was light, HSI score of 3-4 was moderate, HIS score of 5-6 was heavy.
[c] Excluded for participants received different interventions and small sample size.
[d] 141 excluded because they could not be matched to company-level questionnaire.
[e] Excluded because participants lacked workplace SC support information.

Table 3. Questionnaire of workplace smoking cessation support.

Chinese version scale	English translation
**戒煙員工**	**Incentive for successful quitters**
提供現金獎賞給成功戒煙員工	Cash
提供假期獎賞給成功戒煙員工	Holidays
提供禮物獎賞給成功戒煙員工	Gifts
公開表揚成功戒煙的同事 (例如舉辦「戒煙頒獎禮」)	Public praises
**額外的戒煙支援服務**	**Support for smoking cessation attempts/ actions**
提供時間給員工參與戒煙工作坊	Paid time off for attending smoking cessation workshops
設立「無煙茶水間」，於公司的茶水間放置免費健康小食/檸檬片供同事享用，鼓勵同事於小休時間到茶水間休息，減少吸煙	Offer free healthy snacks in the pantry to replace smoking breaks
組織「無煙分享小組」，邀請成功戒煙的同事以及有興趣戒煙的吸煙員工一起定期聚會，分享戒煙的感受以及戒煙的經驗	Organize sharing sessions on successful quitting experiences
▪月/首月現金津貼正在戒煙的同事和戒煙 Buddy 參加運動班，如做 Gym, Funky dance, Yoga 等	Offer reimbursement for joining exercise program/classes
**落實實質的無煙環境政策**	**Smoke-free environment support**
利用電郵、告示板、貼海報、放置宣傳品等方法讓員工了解和遵守公司政策及提供戒煙資訊	Email, notice, posters, other circulation on smoke-free information
制定「無煙月」，將員工電腦桌面或螢幕保護(screen saver)設定為「禁煙標誌」	Set desktop/ screen saver as “smoke-free logo”
公司與同事簽定無煙約章或舉行誓師大會	Sign “Smoke-free contact” with employees
成立「無煙工作間」委員會，鼓勵員工提出不同意見，一起創造無煙工作間，增加員工歸屬感	Set up “Smoke-free workplace” committee
於公司▪張貼一些成功戒煙個案分享	Poster on successful quitting cases
舉辦戒煙口號比賽	Organize “smoke-free slogan” competition

Employers were asked if these supports were provided.

Table 4. Characteristics of employers who have attended interview.

Case No.	Sex	Age (years)	Company industry
1	F	32	Healthcare support
2	M	32	Property management
3	F	32	Construction
4	F	28	Property management
5	F	/	Property management
6	M	38	Property management
7	M	39	Information technology
8	M	35	Sales
9	M	37	property management

© 2022 Guo Z. et al.
